# Sex Differences in the Relationship between Asthma and Overweight in Dutch Children: a Survey Study

**DOI:** 10.1371/journal.pone.0077574

**Published:** 2013-10-17

**Authors:** Maartje Willeboordse, Donna L. C. M. van den Bersselaar, Kim D. G. van de Kant, Jean W. M. Muris, Onno C. P. van Schayck, Edward Dompeling

**Affiliations:** 1 Department of Pediatric Respiratory Medicine, School for Public Health and Primary Care (CAPHRI), Maastricht University Medical Center (MUMC), Maastricht, The Netherlands; 2 Department of Family Medicine, CAPHRI, MUMC, Maastricht, The Netherlands; University of Tübingen, Germany

## Abstract

**Objective:**

Obesity has been identified as a risk factor for asthma in children. However, in the Netherlands, the obesity prevalence is rising while the asthma prevalence in children is stabilising. The aim of this study is to clarify the association between asthma and Body Mass Index (BMI) in children and whether this association is influenced by sex.

**Study Design:**

Parents of 39,316 children (6-16 years) in the south of the Netherlands were invited to complete an online questionnaire on respiratory symptoms, anthropometric variables and several potential confounding factors for asthma and obesity (including sex, birth weight and breastfeeding). Data was analysed by multivariable logistic regression models and an ordinal regression model.

**Results:**

The response rate was 24% (n boys= 4,743, n girls= 4,529). The prevalence of asthma, overweight and obesity was 8%, 15% and 2% respectively. Body mass index - standard deviation Score (BMI-SDS) was related to current asthma (adjusted OR: 1.29; 95%CI: 1.14-1.45, p≤0.001). When stratified for sex, asthma and BMI-SDS were only related in girls (Girls: adjusted OR: 1.31; 95%CI: 1.13-1.51, p≤0.001. Boys: adjusted OR: 1.01; 95%CI: 0.91-1.14, p=0.72).

**Conclusions:**

The positive association between BMI-SDS and asthma is only present in girls, not boys. Future studies into obesity and asthma should correct for sex in their analyses.

## Introduction

Worldwide, there has been an epidemic increase in the prevalence of overweight and obesity, both in adults and children [1]. This alarming increase is also present in the Netherlands, where it has been demonstrated that more than 13% of children aged 5-16 are overweight, with the highest prevalence in girls (17%) [2]. The increase in overweight during the last few decades has coincided with a steep increase in the worldwide asthma prevalence [3]. Therefore, a relationship between overweight and asthma has been hypothesized [4]. A recent meta-analysis by Chen et al. reported a relative risk (RR) of 1.19 (95%CI: 1.03-1.37) for children who are overweight and a RR of 2.02 (95%CI: 1.16-3.50) for children who are obese of developing asthma in the future [5]. Several possible biological underlying mechanisms have been proposed describing the potential effect of obesity on the pulmonary physiology, inflammatory mechanisms, and/or co-morbidities such as gastro-oesophageal reflux [6,7].

In the Netherlands, the initial increase in asthma prevalence has stabilized or even declined since the late 1990s, especially amongst children [3,8]. Yet, since the prevalence of overweight and obesity is still rising in the Netherlands, the hypothesized asthma-obesity relationship can be questioned. Moreover, various aspects of the putative relationship are still unclear. For example, the influence of sex as a potential effect modifier on the asthma-obesity relationship in children has not yet been determined. In adults, it has been demonstrated that the asthma-obesity relationship is more strongly associated with the female sex [9]. However in children, results are conflicting [4,5,10,11]. Moreover, several studies have suggested that obese asthmatics have more severe asthmatic symptoms and more frequent inhaled corticosteroid (ICS) use [7,12]. However, these findings have not been uniformly reported in childhood [7,12,13]. This study was designed to investigate the relationship between asthma and Body Mass Index – Standard Deviation Score (BMI-SDS) and to evaluate the potential influence of sex in Dutch children.

## Methods

### Study design and population

**Figure 1:**
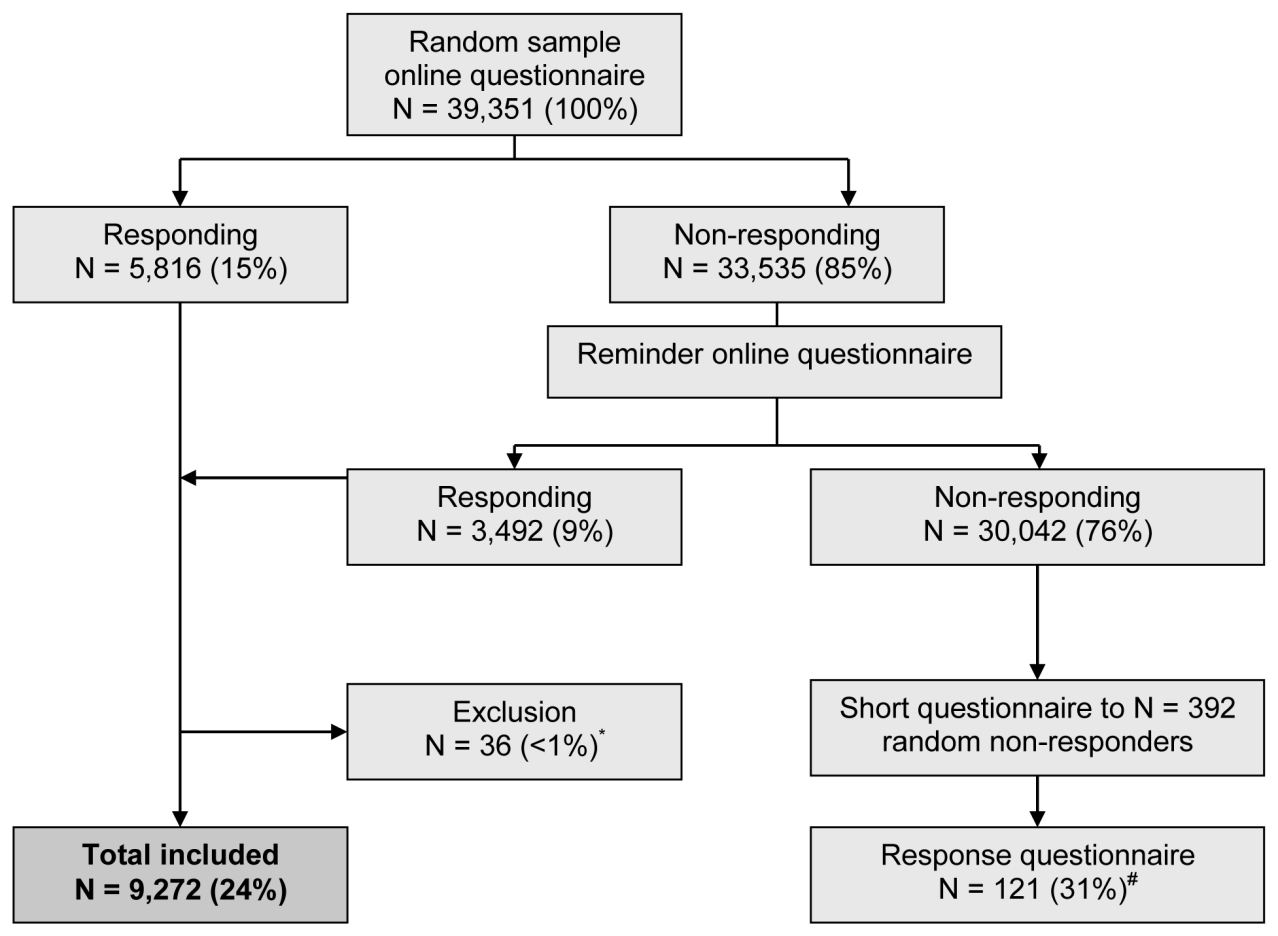
Study flow diagram. * Percentage of the total group of responders. ^#^ Response percentage of the random group of non-responders that received the additional questionnaire.

This cross-sectional study was performed in South Limburg, a province of the Netherlands. Parents of children aged 6-16 years (N=39,351) living in South Limburg received an online self-administered questionnaire about the health of their children via the local authorities. The questionnaire was sent out in May 2010. After three weeks the non-responders received a reminder (Figure 1). A random sample of 400 parents in the non-responding group received a short questionnaire about the main outcome parameters, in order to compare the responding and non-responding group. The study was approved by the Ethics Committee of the Maastricht University Medical Centre (NL28214.068.09 / MEC 09-2-088).

### Main outcome parameters

The online questionnaire included questions about anthropometric variables, the International Study of Asthma and Allergy in Children (ISAAC) questionnaire, and questions relating to potential confounding factors for asthma and the asthma-BMI-SDS relationship [14,15]. 

#### Anthropometrics

Parental reports of the body weight (to the nearest 0.1 kg) and length (to the nearest cm) of the children was used. The BMI-SDS was calculated using the Lambda Mu Sigma method of Cole et al., with the reference values of the fourth national Dutch growth study (1997) [16,17]. BMI-SDS was used as a continuous outcome variable. For illustrative purposes, children were also distributed into weight categories. Underweight was defined as a BMI-SDS <-1.8 and normal weight as a BMI-SDS ≥-1.8 and <1.1. A child was defined as overweight with a BMI-SDS ≥1.1 and <2.3 and as obese with a BMI-SDS ≥2.3 [18].

#### Asthma

Based on the ISAAC questionnaire, children were defined as having current asthma if the parent/caretaker reported that the child had ever been diagnosed with asthma by a health practitioner and if at least one of the following criteria had been met in the last twelve months: 1. At least one episode of wheezing or whistling in the chest; 2. A dry cough at night, apart from a cough associated with a cold or chest infection, for at least one night a week; 3. Use of inhalation medication (bronchodilators and/or ICS).

### Confounders

Based on previous research, the parameters sex of the child and age (in years) were chosen as *a priori* confounders for the asthma-BMI-SDS relationship [14,15]. Other potential confounders which were addressed in the questionnaire included: highest educational level of parents, family history of asthma (defined as asthma in at least one first-degree family member), active smoking by the child, second-hand smoking (defined as at least one family member smoking ≥once a week in the presence of the child), maternal smoking during pregnancy, breastfeeding duration (categories used: 0 months, ≤2 months, 3-4 months, 5-6 months and >6 months), number of siblings (0,1,2,3, ≥4), ethnicity (defined as non-native when at least one of the parents was not born in the Netherlands), attendance at a day care centre before the age of 5 years, maternal antibiotic use during pregnancy, birth weight (categories used: <1500 gram, 1500-1999 gram, 2000-2499 gram, 2500-3499 gram and >3500 gram), presence of hairy pets at home since birth, growing up on a farm, and puberty (based on ≥10.7 years for girls and ≥11.5 for boys as defined by De Muinich-Keizer [19]). 

### Statistical analysis

Data were analysed with SPSS 20.0 (SPSS inc., Chicago IL, USA). Baseline characteristics and the characteristics of the responding and non-responding group were compared by using an unpaired t-test for continuous variables (as all variables were normally distributed) and a chi-square test for categorical variables. Due to the electronic structure of the questionnaire there were no missing data, except for several cases with unrealistic values in the BMI-SDS range (Figure 1). 

#### Primary model

A multivariable logistic regression model was used to evaluate the relationship between BMI-SDS and current asthma. In a pre-analysis we tested the significance of the interaction terms ‘age by sex’ and ‘puberty by sex’ as well as the following terms by BMI-SDS: ‘sex’, ‘age’, ‘puberty’, ‘highest educational level’, ‘birth weight’, ‘passive smoking’, ‘smoking during pregnancy’ and ‘ever having breastfeeding’. In the final regression model, BMI-SDS, the *a priori* confounders (‘sex’ and ‘age’) and significant interaction terms were added by the enter procedure; other confounders (those mentioned above) were added by the Forward Likelihood Ratio (LR) procedure if the p-value was ≤0.10. Separate models were created for the confounders ‘age’ (in years) and ‘puberty’ to avoid multicollinearity. Results are presented with the confounder age unless stated otherwise. For all significant interaction terms the Odds Ratio’s (OR) between asthma and BMI-SDS were additionally calculated by using stratification. We adjusted for multiple testing with the Bonferroni correction. All p-values in the primary analyses are presented as Bonferroni corrected values. Results are presented as adjusted OR and their 95% confidence intervals (95%CI). 

#### Sub analyses

Within the group of children with asthma, we investigated the effect of BMI-SDS on wheezing in the previous 12 months (categories: never, 1-3 episodes, 4-12 episodes, >12 episodes) and dry cough at night in the previous 12 months (categories: never, <1 night per month, >1 night per month and <1 night per week, >1 night per week) by using an ordinal regression model. We included the following selection of potential confounders: ‘age’ (in years), ‘sex’, ‘family history of asthma’, ‘passive smoking’ and ‘educational level of the parents’. 

The effect of BMI-SDS on the use of ICS (yes/no) was calculated using a logistic regression model. We included the following confounders *a priori* using the enter method: ‘BMI-SDS’, ‘sex of the child’ and ‘age’ (in years). The following confounders were added by using the forward LR procedure: ‘family history of asthma’, ‘passive smoking’ and ‘educational level of the parents’. 

#### Sample size calculation

A sample size calculation was performed to identify the number of participants needed to detect an OR of 1.94 between asthma and overweight in children, as described by Rodriguez et al. [20]. The Kelsey formula for observational epidemiology was used, taking an alpha error of 5%, a power of 80% and a ratio of exposed (overweight/obese) vs. unexposed (normal weight) subjects of 1:5. If assumed that the asthma prevalence among the normal weight subjects would be 7%, then 1,228 participants were determined to be sufficient to detect an asthma-BMI-SDS association in this population.

## Results

### Response to questionnaire

A flow chart of the study inclusion is provided in Figure 1. After a reminder, the response rate to the questionnaire was 24% (N=9,308). In total, 9,272 study participants were included, as 36 children were excluded because of unrealistic values in the BMI-SDS range. The response to the questionnaire in the selected non-responding group was 31% (N=121) (Figure 1). The responders and non-responders groups did not differ regarding baseline characteristics such as asthma prevalence, BMI-SDS and asthma in a first-degree family member (table S1 in File S1).

### Baseline characteristics

The prevalence of current asthma in the total population was 7.6% (n=709). In total, 15.2% (n=1,412) of the children were overweight and 2.4% (n=225) of the children were obese (table 1). The mean age of the participants was 10.9 years (range 5.6 - 17.3 years). The majority of the children were native Dutch (n=7,799, 84%). Nearly half of the asthmatic children suffered from atopic symptoms such as hay fever or eczema. Three-quarters of the asthmatic children experienced at least one wheezing episode in the last year and 95% of the asthmatics used bronchodilators. Compared to the non-asthmatic children, asthmatic children were more likely to have a high BMI and BMI-SDS, parents with a low educational level, a family history of asthma and were more often boys (table 1). Additional baseline characteristics can be found in the table S2 in File S1. 

**Table 1:** Baseline characteristics.

**Characteristics**	**Total group (n=9,272)**	**Asthma**	
		**Yes (n=709)**	**No (n=8,563)**
Mean age, years(Range)	10.9 (5.6-17.3)	11.1 (6.1-17.3)	10.9 (5.6-17.3)
Male sex, N(%)*	4,743 (51.2)	417 (58.8)	4,326 (50.5)
Height, cm(Range)	147 (100-195)	147 (110-191)	147 (100-195)
Weight, kg(Range)	39.4 (15.0-107.0)	40.5 (15.9-106.0)	39.3 (15.0-107.0)
BMI, kg/m^2^(Range)*	17.6 (10.4-44.4)	18.1 (11.6-37.5)	17.5 (10.4-44.4)
BMI-SDS(Range)*	-0.03 (-5.41-4.62)	0.15 (-3.95-3.30)	-0.04 (-5.41-4.62)
Underweight, N(%)	694 (7.5)	44 (6.2)	650 (7.6)
Normal weight, N(%)	6,941 (74.9)	511 (72.1)	6,430 (75.1)
Overweight, N(%)	1,412 (15.2)	121 (17.1)	1,291 (15.1)
Obesity, N(%)*	225 (2.4)	33 (4.7)	192 (2.2)
Non native Dutch children, N(%)	1,473 (15.9)	101 (14.2)	1,372 (16.0)
Mothers with a low educational level, N(%)*	1,773 (19.1)	175 (24.7)	1,598 (18.7)
Fathers with a low educational level, N(%)*	1,898 (20.5)	165 (23.3)	1,733 (20.2)
Family history of asthma, N(%)*	2330 (25.1)	389 (54.9)	1941 (22.7)
Wheezing episodes			
*No wheezing periods^#^, N(%)^*	8391 (90.5)	178 (25.1)	8213 (95.9)
*1-3 wheezing periods* ^*#*^ *, N(%*)*^*	564 (6.1)	308 (43.4)	256 (3.0)
*4-12 wheezing periods* ^*#*^ *, N(%*)*^*	237 (2.6)	160 (22.6)	77 (0.9)
*>12 wheezing periods* ^*#*^ *, N(%*)*^*	80 (0.9)	63 (8.9)	17 (0.2)
Ever had hay fever, N(%)*	1456 (15.7)	327 (46.1)	1129 (13.2)
Ever had eczema, N(%)*	2756 (29.7)	398 (56.1)	2358 (27.5)
Medication use			
*Bronchodilator^#^, N(%)^*	621 (6.7)	603 (85.0)	18 (0.2)
*Inhaled corticosteroids^#^, N(%)^*	441 (4.8)	423 (59.7)	18 (0.2)
*Other asthma medication^#^, N(%)^*	104 (1.1)	84 (11.8)	20 (0.2)

Legend:

(%): Percentage of the characteristic within each presented column of the table (i.e. % of subjects with the male sex in the total group, asthma group, no asthma group respectively).

* *p* <0.05*, significant* *differences* *between* *asthma and no-asthma* *group.*

# During the previous 12 months.

^ p-values were not calculated, as wheezing and medication usage were part of the asthma definition.

Abbreviations: BMI= Body Mass Index. SDS = Standard Deviation Score.

### Primary analyses

**Figure 2:**
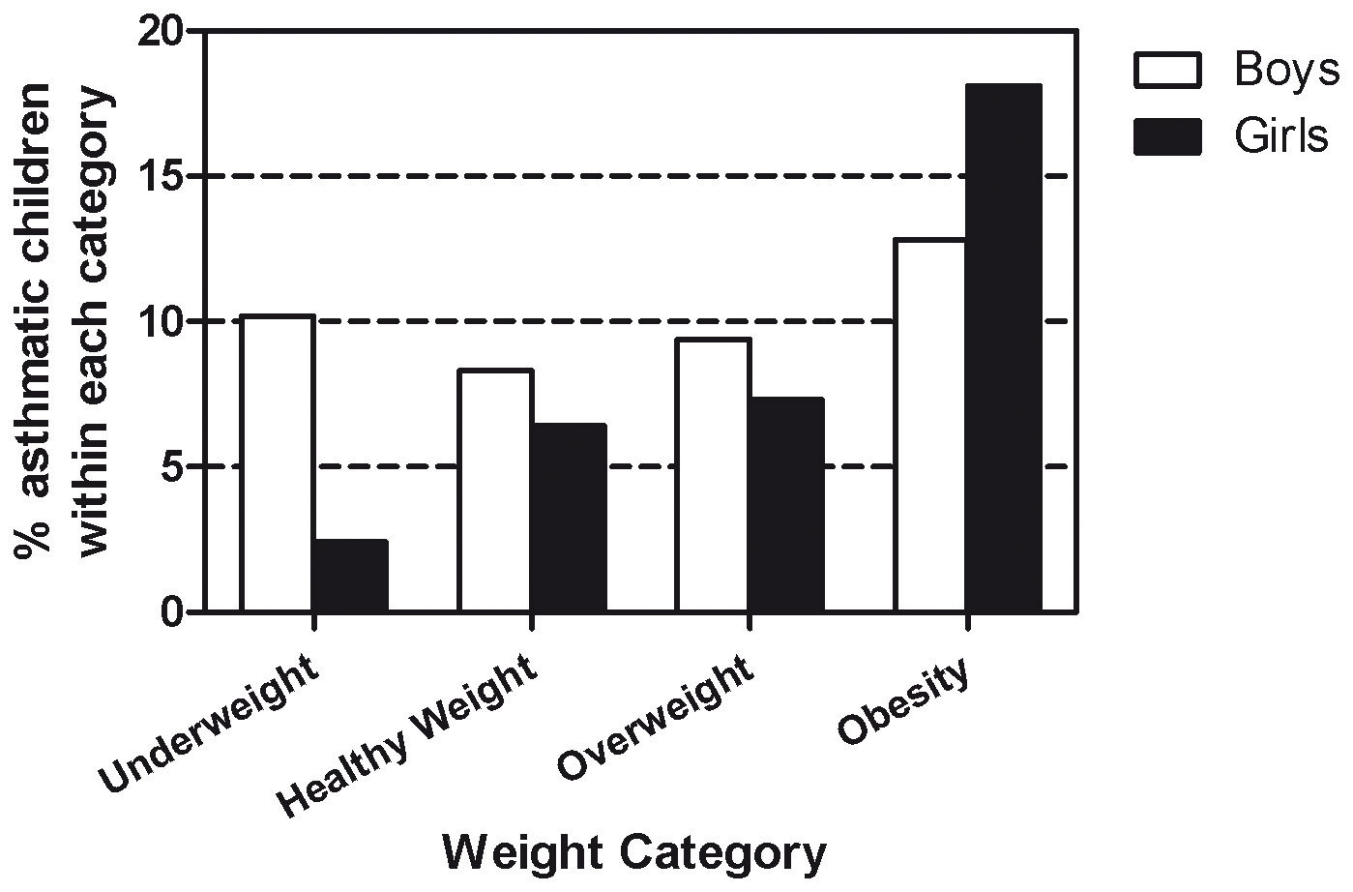
Percentage of boys and girls with asthma within different weight categories.* * Cut-off values for weight categories were chosen for illustrative purposes, analyses were performed with BMI-SDS as a continuous variable.

After adjustment for significant confounding factors, BMI-SDS was positively associated with asthma (adjusted OR: 1.29; 95%: 1.14-1.45, p≤0.001) (table 2, Figure 2). Stratification for sex revealed that the relationship between BMI-SDS and asthma was significant in girls but not in boys (Girls: adjusted OR: 1.31; 95%CI: 1.13-1.51, p≤0.001. Boys: adjusted OR: 1.01; 95% CI: 0.91-1.14, p=0.72) (Figure 2). Neither age nor puberty were significant confounders for the asthma-obesity relationship in both boys and girls. In addition to a high BMI-SDS in girls, several other factors were also found to predict the prevalence of asthma, including male sex, asthma in a first-degree family member and parents with a low or average educational level as opposed to a high educational level (table 2). 

**Table 2:** Odds Ratios (OR) of having current asthma.

	**Odds ratio (95% CI)**	**Odds ratio (95%CI) stratified for girls**	**Odds ratio (95%CI) stratified for boys**
BMI-SDS	1.29 (1.14-1.45)^*^	1.31 (1.13-1.41) ^*^	1.02 (0.91-1.14)
Sex ^1^	1.48 (1.26-1.75) ^*^	-	-
Age in years	1.01 (0.99-1.05)	1.03 (0.98-1.07)	1.01 (0.97-1.05)
Family history of asthma ^2^	0.25 (0.21-0.29) ^*^	0.21 (0.17-0.27) ^*^	0.27 (0.22-0.33) ^*^
Ethnicity ^3^	1.26 (0.99-1.60)	-	1.32 (0.96-1.82)
Presence of hairy pets in the household	1.15 (0.98-1.35)	-	-
Low educational level ^4^	1.50 (1.17-1.92) ^*^	1.40 (0.95-2.04)	1.45 (1.05-2.00)
Average educational level ^4^	1.31 (1.10-1.56) ^*^	1.31 (0.99-1.73)	1.26 (1.00-1.58)
BMI-SDS x Sex	0.80 (0.67-0.91) ^*^	-	-
Birth weight ≤1500 gram^5^	1.08 (0.60 - 1.96)	1.77 (0.80-3.94)	0.66 (0.26-1.66)
Birth weight 1500-1999 gram^5^	0.98 (0.61 - 1.57)	1.13 (0.57-2.26)	0.87 (0.45-1.68)
Birth weight 2000-2499 gram^5^	1.15 (0.87-1.52)	1.16 (0.76-1.77)	1.1 (0.75-1.61)
Birth weight ≥3500 gram^5^	0.87 (0.72 - 1.04)	0.99 (-0.73-1.33)	0.80 (0.63-1.11)
Birth weight ≤1500 gram x BMI-SDS	0.71 (0.50 - 0.99)	0.88 (0.48-1.60)	0.61 (0.39-0.96)
Birth weight 1500-1999 gram x BMI-SDS	0.68 (0.48-0.97)	0.75 (0.45-1.22)	0.61 (0.37-0.99)
Birth weight 2000-2499 gram x BMI-SDS	1.09 (0.87-1.36)	1.14 (0.78-1.65)	1.05 (0.79-1.39)
Birth weight ≥3500 gram x BMI-SDS	1.01 (0.87-1.17)	0.89 (0.69-1.14)	1.07 (0.89-1.29)

Legend:

1 0=female, 1=male

2 0= no family history of asthma, 1= family history of asthma

3 0= native ethnicity, 1= non-native ethnicity

4 Opposite to a high educational level

5 Opposite to a birth weight of 2500-3499 gram

* p <0.05 after bonferroni correction

OR could not be calculated because term could not be entered in the model (for terms including sex) or was not entered in the model by Forward LR method.

Abbreviations: BMI-SDS= Body Mass Index - Standard Deviation Score.

### Sub analyses

In the subgroup of asthmatic children, BMI-SDS was not significantly related to asthma parameters, including frequency of wheezing (adjusted OR: 0.92; 95%CI: 0.83-1.03, p=0.16), frequency of dry cough at night (adjusted OR: 1.01; 95%CI: 0.89-1.15, p=0.88), and ICS use (adjusted OR: 0.95; 95%CI: 0.83-1.10, p=0.51).

## Discussion

In this study, BMI-SDS was associated with current asthma in 6 to 16 year old children. After stratification for sex, a significant relationship between BMI-SDS and asthma was revealed only in girls. In children with asthma, BMI-SDS was not related to frequency of wheezing, dry cough at night and use of ICS. 

The OR of 1.29 between asthma and BMI-SDS in our study agrees closely with a recent meta- analysis that studied the relationship between asthma and BMI in children. In this meta-analysis, a pooled RR of incident asthma in overweight children of 1.19 (95%CI: 1.03-1.37) and obese children of 2.02 (95%CI: 1.16-3.50) was calculated [5]. Moreover, we demonstrated that asthma prevalence was related to several other factors including male sex, family history of asthma, and parents with a low or average educational level as opposed to a high educational level. These findings are in agreement with factors found in previous studies [15]. 

We verified that asthma prevalence in general was higher amongst boys than girls. When stratified for sex, BMI-SDS was not related to current asthma in boys, but there was a significant positive relationship between BMI-SDS and asthma prevalence in girls. This finding is in line with a meta-analysis performed by Beuther et al. in adults, in which the asthma-obesity relationship was stronger in woman than in men (men vs. woman: OR: 1.46; 95%CI: 1.05-2.02 vs. 1.68; 95%CI: 1.45-1.94 ) [21]. In contrast, studies in children have so far shown inconsistent results. One meta-analysis concluded that obese boys had a significantly higher risk of incident asthma than obese girls (boys vs. girls: RR: 2.47; 95%CI: 1.57-3.87 vs. 1.25; 95%CI: 0.51-3.03) [5]. However, this conclusion was based on limited studies. In several other reviews, it was argued that the association of BMI is generally stronger in girls than in boys, especially after puberty [4,10,11]. Regardless of the direction of these findings, it is crucial that future studies on obesity and asthma should correct for sex as it is a significant confounder. 

In adults, several hypotheses are described for the sex-specific asthma-obesity relationship. The most commonly used hypothesis focuses on hormonal differences; in particular, the role of oestrogen in the female sex is often described in literature [9]. Although the exact mechanisms are unclear, oestrogen affects lung function and inflammatory processes via various pathways [22]. In children, it is often hypothesized that oestrogen in the adipose tissue causes an early menarche in girls and a delayed puberty in boys [23]. An early menarche is related to a higher risk of developing asthma and a stronger association between BMI and asthma severity [23,24]. However, in our study we found no effect of either age or puberty (as defined by cut-off values based on age [19]) on the asthma-obesity relationship in both boys and girls. 

In addition to the oestrogen-hypothesis, it has been suggested that adipose derived hormones such as leptin and adiponectin play a significant role in the asthma-obesity relationship [12]. Excessive adipose tissue is related to increased leptin levels and decreased adiponectin levels, which results in an increased leptin-adiponectin ratio. It has been hypothesized that high serum leptin levels precede airway inflammation, an important asthma characteristic [12]. In asthmatic children, high serum leptin levels are related to the female sex [9,25]. Ali Assad et al. demonstrated in a recent review that the association between serum leptin and asthma prevalence is stronger among specific population subgroups such as pre-pubertal boys and peri-pubertal or post-pubertal girls [26]. However, it is not known exactly how these possible sex-interactions work, and a greater understanding of the role of sex in the leptin-adiponectin ratio is necessary. 

A third possible hypothesis for our findings is that girls have a genetic predisposition for the asthma-obesity phenotype. Asthma prevalence is related to the chromosomal gene Cysteinyl Leukotriene Receptor-1 (CYSLTR1) and it has been suggested that the underlying genetic pathogenesis of asthma is sex-specific [27]. In addition, several genes have been related to the asthma-obesity phenotype. Su et al. found that the obesity-related gene Insuline Induced Gene 2 (INSIG2) is related to childhood asthma and that there is an interaction between the inflammatory inter leukine 4 receptor alpha gene, obesity-related INSIG2 gene and anti-oxidative glutathione S transferase pi gene [28]. Future studies should investigate whether asthma-obesity related genes are associated with sex-specific asthma-related genes such as CYSLTR1. 

Another hypothesis is of a more methodological origin. It has been reported that women are more likely to report asthma-like symptoms than men. Under-diagnosed asthma is more frequent amongst men than women [29]. Moreover, there seems to be a sex-specific misclassification of obesity in adults. A study in Mexican adults suggested that the use of self-reported BMI underestimated the prevalence of obesity in men, and therefore obscured the relationship between obesity and asthma in men [30]. In our study, anthropomorphic measures used to calculate BMI-SDS were parental-reported, and it is known that parents can (subconsciously) treat and assess children differently because of different body image attributions in boys and girls [31]. 

Typical characteristics of the obese-asthmatic child are less asthma control and more frequent ICS use [7,12]. In our study, asthmatic children with an elevated BMI-SDS did not experience more asthmatic symptoms (wheeze, dry cough) than asthmatic children with a normal BMI-SDS. For future studies it is of interest to incorporate objective measurements of asthma such as lung function, airway inflammation markers or the presence of co-morbidities in order to elucidate the asthmatic-obese phenotype [12].

An important strength of our study is that the respondents of our study are a representative sample of the population. The prevalence of asthma of 7.6% in this study is consistent with the average Dutch childhood asthma prevalence of 7.2% [3]. Also, the prevalences of overweight and obesity in our population are comparable to the Dutch average [2]. Moreover, a comparison of the responders and non-responders on the most important outcome measures revealed no significant differences. Another advantage of this study design is that there are hardly any missing data due to the electronic structure of the questionnaire. As this study has a high number of study participants and a representative study population, there is strong evidence that our conclusions can be extrapolated to the population level. 

Some challenges for future studies need to be mentioned. Firstly, as in most large-scale epidemiological studies, asthma diagnosis was not defined by objective lung function measurements [4]. Large-scale studies that include objective measures of asthma could add useful information to this study area. Secondly, future longitudinal studies are necessary as cross-sectional studies cannot define a causal relationship between overweight and asthma. As the exact mechanisms between asthma and obesity are still unknown, weight loss studies or longitudinal cohort studies could provide more information about the inflammatory pathways and pulmonary physiology that could contribute to the asthma-obesity phenotype [7,12]. Thirdly, participants with less formal education are in general underreported in large-scale cross-sectional studies [32]. In this study, 20% of the participants had a low educational level, as opposed to an average low educational level of 35% in the region [33]. Nevertheless, as the educational level did not interact with BMI-SDS, it is unlikely that the educational level has influenced the outcomes of this study. Future studies should focus on new study methods in which underreporting of people with less formal education could be diminished. Fourthly, a total of 36 participants were excluded because of unrealistic BMI values. As these participants represented <0.5% of our total study sample, it is not likely that exclusion of these participants confounded our results. However, future studies could prevent this by adding a pop-up warning in their online questionnaire in case an unrealistic combination of length and weight is entered. 

In conclusion, we demonstrated a small but significant positive relationship between BMI-SDS and asthma in girls, but not in boys. It is probable that several underlying mechanisms are responsible for the sex differences in the asthma-obesity relationship. Therefore, it is crucial that future studies into obesity and asthma differentiate between males and females. The role of oestrogen, leptin, adipokine, genes and epigenetics should be further investigated, as these features might play an important role in the relationship between sex, asthma and obesity. 

## Supporting Information

File S1 **Supporting Files**.
Table S1, Characteristics of the responder and non-responder group. Legend: # During the previous 12 months. * p <0.05, significant differences between responder and non-responder group. Abbreviations: BMI= Body Mass Index, BMI-SDS = BMI - Standard Deviation Score. Table S2, Additional baseline subject characteristics. Legend: * p <0.05, significant differences between asthma and no-asthma group.
(DOC)
