# Supplementary material for: Sex Differences in the Relationship between Asthma and Overweight in Dutch Children: a Survey Study
Source: PLoS One. 2013 Oct 17;8(10):e77574. doi: 10.1371/journal.pone.0077574 (PMC3804131; doi:10.1371/journal.pone.0077574)
Supplement: File S1 — Supporting Files. Table S1, Characteristics of the responder and non-responder group. Legend: # During the previous 12 months. * p <0.05, significant differences between responder and non-responder group. Abbreviations: BMI= Body Mass Index, BMI-SDS = BMI - Standard Deviation Score. Table S2, Additional baseline subject characteristics. Legend: * p <0.05, significant differences between asthma and no-asthma group. (DOC) [file pone.0077574.s001.doc]

**File S1**

**Table S1. Characteristics of the responder and non-responder group.**

Legend:

# During the previous 12 months

* p <0.05, significant differences between responder and non-responder group.

Abbreviations: BMI= Body Mass Index, BMI-SDS = BMI - Standard Deviation Score.

| **Variable** | **Responder group**  **(n= 9,272)** | **Non-responder group**  **(n = 121)** |
| --- | --- | --- |
| Male gender, N(%) | 4,743 (51.2) | 61 (50.4) |
| Mean age, years(SD) | 10.9 (2.7) | 11.4 (2.7) |
| BMI (SD) | 17.6 (3.2) | 18.0 (3.4) |
| BMI-SDS | -0.03 | 0.03 |
| Current asthma, N(%) | 709 (7.6) | 4 (3.3) |
| Wheezing^#^, N(%) | 915 (9.9) | 12 (9.9) |
| Dry cough at night^#^, N(%) | 1,554 (16.8) | 20 (16.5) |
| Family history of asthma, N(%) | 2,330 (25.1) | 26 (21.5) |
| Breastfeeding, N(%) | 3,765 (40.6) | 46 (38.0) |
| Itchy rash^#^, N(%) | 1,131 (12.2) | 15 (12.4) |

**Table S2. Additional baseline subject characteristics.**

| **Characteristics** | **Total group** | **Asthma** | |
| --- | --- | --- | --- |
|  |  | **Yes** | **No** |
| Study population, N(%) | 9,272 | 709 (7.6) | 8,563 (92.4) |
| Active smoking, N(%) | 82 (0.9) | 8 (1.1) | 74 (0.9) |
| Passive smoking, N(%) | 2,736 (29.5) | 230 (32.4) | 2,506 (29.3) |
| Maternal smoking during pregnancy, N(%)* | 1,245 (13.4) | 116 (16.4) | 1,129 (13.2) |
| Use of antibiotics during pregnancy, N(%) | 457 (4.9) | 46 (6.5) | 411 (4.8) |
| Growing up at a farm, N(%) | 100 (1.1) | 6 (0.8) | 94 (1.1) |
| Breastfeeding, N(%)* | 5,371 (57.9) | 380 (53.6) | 4,991 (58.3) |
| Duration ≥0 and <2 months, N(%) | 1,507 (28.1) | 120 (31.6) | 1,387 (27.8) |
| Duration ≥2 and ≤6 months, N(%)* | 2,925 (54.5) | 197 (51.8) | 2,728 (54.7) |
| Duration >6 months, N(%) | 939 (17.5) | 63 (16.6) | 876 (17.6) |
| Birth weight, N(%) |  |  |  |
| *<1500 gr* | 160 (1.7) | 17 (2.4) | 143 (1.7) |
| *1500-1999 gr* | 271 (2.9) | 24 (3.4) | 247 (2.9) |
| *2000-2499 gr* | 764 (8.2) | 69 (9.7) | 695 (8.1) |
| *2500-3500 gr* | 4,885 (52.3) | 376 (53.0) | 4,509 (52.7) |
| *>3500 gr* | 3,025 (32.6) | 211 (29.8) | 2,814 (32.9) |
| Birth delivery, N(%) |  |  |  |
| *Natural birth* | 7,990 (86.2) | 605 (85.3) | 7,385 (86.2) |
| *Caesarean section planned* | 453 (4.9) | 37 (5.2) | 416 (4.9) |
| *Caesarean section acute* | 764 (8.2) | 61 (8.6) | 703 (8.2) |

Legend:

* p <0.05, significant differences between asthma and no-asthma group.
